# Antioxidant, Antibacterial, and Dental Bond Strength Performance of *Picea orientalis* Resin

**DOI:** 10.3390/molecules31142529

**Published:** 2026-07-21

**Authors:** Özge Mimir Özgenç, Tugba Serin Kalay, Ülkü Zeynep Üreyen Esertaş, Sevgi Kolaylı

**Affiliations:** 1 Department of Restorative Dentistry, Faculty of Dentistry, Üsküdar University, İstanbul 34662, Türkiye; ozge.mimir@uskudar.edu.tr; 2 Department of Restorative Dentistry, Faculty of Dentistry, Karadeniz Technical University, Trabzon 61080, Türkiye; 3 Department of Microbiology, Faculty of Medicine, Ağrı İbrahim Çeçen University, Ağrı 04100, Türkiye; uzesertas@agri.edu.tr; 4 Department of Chemistry, Faculty of Science, Karadeniz Technical University, Trabzon 61080, Türkiye; 5 Faculty of Natural Sciences and Agriculture, Nakhchivan State University, Nakhchivan AZ7012, Azerbaijan

**Keywords:** *Picea orientalis*, resin, antioxidant, antibacterial, dental cavity

## Abstract

The objective of this study was to evaluate the antioxidant and antibacterial profiles of *Picea orientalis* (Oriental spruce) resin extracts collected from three distinct geographical sites (Artvin, Borçka, and Çaykara) within the Eastern Black Sea region of Türkiye. Additionally, it aimed to assess the viability of these extracts as natural antibacterial agents against cariogenic pathogens and to determine their subsequent effect on dental bond strength performance. Quantitative phytochemical analysis of the ethanolic extracts included the determination of total phenolic content, coupled with individual phenolic profiling by HPLC-PDA. The antioxidant potential was assessed through DPPH and FRAP assays. Additionally, the antibacterial activity against a panel of oral and systemic pathogens was evaluated using agar well diffusion. Furthermore, to evaluate mechanical performance, composite bond strengths were tested on 180 dentin specimens using both self-etch and etch-and-rinse adhesive strategies, following cavity pre-treatment with a control, chlorhexidine, or the resin extracts. The results revealed that the resin from the Artvin region exhibited the highest phenolic content, characterized by an abundance of trans-cinnamic, caffeic, and ferulic acids, which correlated with their superior antioxidant capacity. Furthermore, while the extracts demonstrated potent inhibitory effects against *Staphylococcus aureus*, their antibacterial activity against *Streptococcus mutans* was found to be comparatively moderate. In bond strength evaluations, the non-aged control group exhibited the highest values (*p* = 0.000); however, no statistically significant difference was observed between the chlorhexidine and resin extract groups (*p* > 0.05). Consequently, the phenolic-rich *P. orientalis* resin represents a promising natural antibacterial alternative for dental applications.

## 1. Introduction

The utilization of natural resins as traditional masticatory agents and for their therapeutic properties has a long history across various cultures [1,2]. Among these materials, resins derived from the Pinaceae family, particularly those obtained from coniferous species such as pine (*Pinus* spp.) and spruce (*Picea* spp.) constitute an important group. In Anatolia, spruce species belonging to the Pinaceae family are commonly known by the local name “Ladin,” reflecting their long-standing cultural and ethnobotanical significance in the region. These resins are classified as secondary metabolites and are secreted as viscous exudates in response to mechanical injury to the tree trunk, where they function as a critical component of the tree’s defense system. Chemically, the exudates are mainly composed of terpenoid compounds, including diterpenes and triterpenes, along with volatile monoterpene-rich essential oils. Upon exposure to air, the volatile fraction, commonly referred to as turpentine, gradually evaporates, while the remaining constituents undergo oxidation and polymerization. This process leads to the formation of a hardened, amorphous solid that is traditionally collected by mechanical scraping from the bark or trunk of the tree [3]. The resulting naturally cured polymeric resin has been used in certain cultures, including Anatolian communities, as non-food chewing gum, largely due to its perceived bioactive and health-promoting properties [4].

*Picea orientalis* (Oriental spruce), a member of the Pinaceae family, is an evergreen conifer reaching 40–50 m in height and naturally distributed in the humid mountainous regions of northeastern Türkiye and the Caucasus, typically at elevations above 1200 m. Its stem resin yields essential oils rich in monoterpene hydrocarbons, mainly limonene, β-pinene, and α-pinene, which are associated with notable biological activities. The resin has been reported to exhibit broad antibacterial and antifungal properties, highlighting *P. orientalis* as a valuable natural resource for traditional medicine as well as pharmaceutical and industrial applications [5,6]. A particularly well-studied natural resin is mastic gum (*P*. *lentiscus*), which has garnered significant scientific interest for its potential benefits in oral health. Contemporary research strongly indicates that mastic gum possesses key antiplaque and antibacterial characteristics [7,8]. A pilot investigation demonstrated that chewing mastic gum was effective in reducing dental plaque formation within the oral cavity [6]. This antiplaque efficacy is largely attributed to the resin’s inhibitory activity against pathogenic oral bacteria crucial for the etiology of dental caries. Specifically, previous studies have reported that mastic gum significantly reduces the levels of acidogenic bacteria, including *S. mutans* and *Lactobacillus* spp., which are major etiological agents in dental caries [9,10]. Furthermore, the antibacterial properties of mastic gum extend to the prevention of biofilm formation. For instance, it was shown that mastic gum resin extract exhibited effects comparable to those of chlorhexidine mouthwash in preventing biofilm accumulation on titanium dental implants. Beyond direct bactericidal effects, it is suggested that mastic gum may contribute to the preservation of dental enamel by influencing salivary pH levels, potentially mitigating the establishment of an acidic environment [10,11]. Given these promising findings, the comprehensive reviews cited highlight mastic gum’s robust potential as a natural adjunct for oral hygiene and maintenance.

Despite the extensive body of research on mastic gum and other well-known natural resins, scientific studies focusing on the resin of *P. orientalis* remain extremely limited. Building upon the documented antibacterial properties of natural resins, the present study aims to address this knowledge gap by providing a comprehensive chemical characterization of oriental spruce (*P. orientalis*) resin and by evaluating its potential therapeutic applications in dentistry.

Therefore, the primary objective of this study was to comprehensively characterize the secondary metabolite profile of *P. orientalis* resin and evaluate its antibacterial efficacy against specific oral pathogens. Additionally, this research aimed to determine the in vitro effect of the resin on the bond strength of dental restorative materials. The novelty of this study lies in being the first to investigate the antibacterial action of *P. orientalis* against these unexamined oral pathogens, alongside its unprecedented application in restorative dentistry. By bridging phytochemical characterization with biomechanical testing, this work generates novel foundational data, highlighting the resin’s potential as a dual-action natural biomaterial to improve oral hygiene and enhance the clinical longevity of dental restorations.

## 2. Results and Discussion

### 2.1. Total Phenolic Contents

In this study, the phenolic composition and antioxidant properties of pine resin samples collected from *P. orientalis* trees at three different locations in the Eastern Black Sea region were evaluated, and the results are summarized in Table 1. The total phenolic content (TPC) of the samples ranged from 378.29 to 389.47 mg GAE/g. Similarly, the total flavonoid content (TFC) varied between 0.09 and 2.97 mg QE/g. While the TPC values of the three samples were found to be nearly identical, statistically significant differences were observed in their TFC levels. The sample obtained from the Artvin region exhibited the highest total phenolic and flavonoid contents.

A previous study examining 13 pine resin samples collected from different geographical regions of Türkiye reported TPC values ranging from 23.19 to 379.44 mg GAE/g [12]. Consistent with our findings, the samples from the Artvin region were identified as having the highest phenolic content. The antioxidant properties of the three mastic gum samples were evaluated using two major analytical techniques. The FRAP assay is based on the reduction of the Fe (III)–TPTZ complex to its Fe (II) form in the presence of antioxidants; thus, higher FRAP values indicate stronger antioxidant capacity. All three samples exhibited comparable antioxidant activity. In a previous study on different pine resin samples, FRAP values were shown to be positively correlated with total phenolic content [13]. The chemical composition of pine resin generally consists of polyphenols and various volatile compounds [14]. The second method used to assess antioxidant activity was the DPPH radical-scavenging assay, which measures the ability of compounds to quench the stable DPPH free radical. In this study, DPPH radical scavenging activity was expressed in terms of the SC_50_ value, which is defined as the extract concentration required to neutralize 50% of the free radicals in the reaction medium. Consequently, a lower SC_50_ value indicates a superior radical scavenging capacity. Among the three evaluated samples, the extract sourced from Borçka demonstrated the most potent DPPH radical scavenging ability, as evidenced by its minimal SC_50_ value. Notably, this strong free radical scavenging performance was further corroborated by the FRAP assay, where the Borçka sample similarly exhibited the highest reducing power. The consistency between these two assays underlines the robust overall antioxidant potential of this specific sample.

### 2.2. Phenolic Composition of the Samples

The phenolic profiles of all three resin samples were analyzed using HPLC–PDA, validated against 26 phenolic standards. The resulting data are summarized in Table 2. Among the 26 phenolic standards analyzed, only six phenolic substances were detected in the samples. Caffeic acid, ferulic acid, and t-cinnamic acid were common to all three samples, whereas *p*-hydroxybenzoic acid, epicatechin, and hesperetin were detected exclusively in the Artvin sample. Gallic acid, *proto*-cathequic acid, chlorogenic acid, syringic acid, *m*-OH benzoic acid, ellagic acid, *p*-coumaric acid, rutin, quercetin, myricetin, apigenin, daidzein, resveratrol, chrysin, rhamnetin, pinocembrin, CAPE and curcumin were found below the limit of detection (LOD). HPLC-PDA chromatograms of all three samples are given in Supplementary Materials S1–S3. The HPLC-PDA analysis further revealed that phenolic acids were present at higher concentrations than flavonoids in all three samples, consistent with the observation that the total phenolic content exceeded the total flavonoid content in each case.

In a study conducted on *Picea abies* (spruce) cones, the presence of astragalin, quercetin, and kaempferol was identified based on a limited set of phenolic standards [15]. Caffeic acid, one of the major bioactive constituents of propolis, was identified as the second most abundant polyphenolic compound in this resin after *trans*-cinnamic acid [16,17].

### 2.3. Antibacterial Activities of the Samples

**Figure 1:**
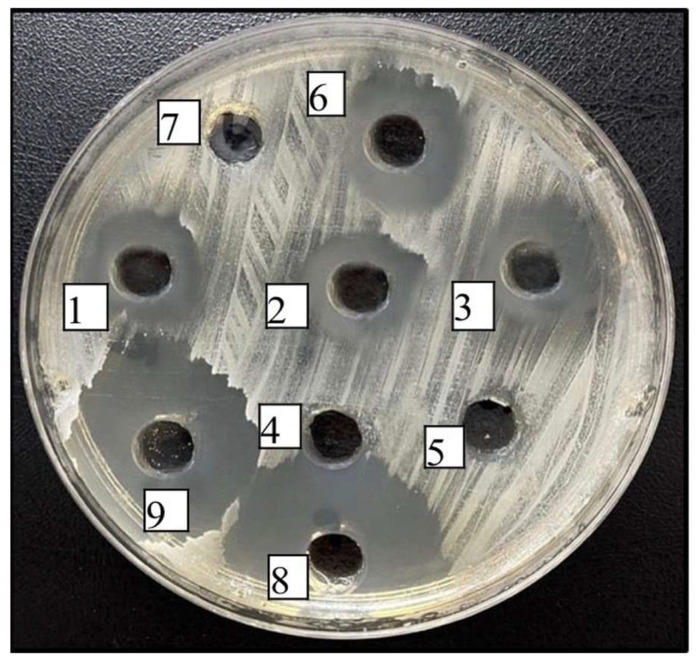
Antibacterial effect on *S. aureus* depending on inhibition zone diameters (1: Çaykara resin extract, 2: Borçka *resin extract*, 3: *Artvin resin extract*, 4: Ferulic Acid, 5: T-cinnamic Acid, 6: Caffeic Acid, 7: Negative Control 8: Ampicillin, 9: Chlorhexidine).

Table 3 and Table 4 present the antibacterial activities of the tested samples along with selected phenolic standards, expressed as minimum inhibitory concentration (MIC) and minimum bactericidal concentration (MBC). In this study, the in vitro antibacterial activities of ethanolic resin extracts were evaluated against three Gram-positive bacteria (*S. aureus*, *S. mutans*, and *E. faecalis*) and a Gram-negative bacterium (*P. aeruginosa*), all of which are clinically relevant to oral and dental health. Examination of the inhibition zone diameters for the three ethanolic resin extracts revealed that all extracts exhibited comparable levels of antibacterial activity, as shown in Table 3 and Table 4. The strongest inhibition was observed against *S. aureus*, whereas the weakest inhibition was detected against *S. mutans* (Figure 1). Furthermore, the extracts showed no inhibitory effect on *E. faecalis* and *P. aeruginosa* strains. The inhibition zone diameters obtained in our study were also compared with those produced by standard reference compounds known to be effective against these microorganisms, including chlorhexidine, ampicillin, gentamicin, and amphotericin *B. aureus* is a Gram-positive bacterium with the potential to cause infections in periodontal tissues and has been implicated in the development of abscesses in certain cases. *E. faecalis*, also a gram-positive species, is frequently isolated from persistent and treatment-resistant endodontic infections; its ability to survive and persist following root canal therapy underscores its clinical significance [18,19].

In contrast, *P. aeruginosa* is a Gram-negative bacterium that is rarely detected in the normal oral microbiota; however, it may contribute to oral infections and wound colonization, particularly in immunocompromised individuals. *S. mutans*, a key member of the oral microbiome, is a Gram-positive bacterium recognized as a major etiological agent in dental caries due to its capacity to metabolize fermentable carbohydrates into organic acids, thereby promoting enamel and dentin demineralization [9,20]. *C. albicans* and *C. parapsilosis* are fungal species capable of causing opportunistic oral candidal infections. In the present study, the resinous pine extract samples did not exhibit any inhibitory activity against either of these fungal strains.

Consistent with our study, mastic gum obtained from *P. lentiscus* has been demonstrated to exhibit antibacterial activity against *S. mutans*, one of the primary cariogenic bacteria responsible for the demineralization of dental enamel and the development of surface caries [10,11].

In our study, the antibacterial and antifungal activities of several major phenolic compounds identified in pine resins, specifically caffeic acid, ferulic acid, and trans-cinnamic acid, were also evaluated. Among these three phenolic standards, caffeic acid demonstrated inhibitory activity against four bacterial strains, excluding the tested fungal species [19,20]. Caffeic acid is one of the major bioactive components of propolis and has been reported in numerous studies as a key contributor to the high antibacterial activity of propolis [21,22,23,24,25]. In addition, the results of the study indicate that the pine resin extracts exhibited selective antibacterial activity, whereas chlorhexidine demonstrated broad-spectrum efficacy against all tested bacterial and fungal strains. Chlorhexidine, a broad-spectrum antibacterial agent, is widely used in dentistry.

No significant differences were observed among the three resin extracts in terms of antioxidant and antibacterial activities. Consequently, the extract from the Artvin region was selected for subsequent cavity disinfectant evaluations.

### 2.4. Dental Bond Strength Performance

Table 5 summarizes the effects of aging and various surface preparation methods on the bond strength of the cavity disinfectants. In the control group, both the etch-and-rinse and self-etch protocols yielded significantly higher bond strength values in non-aged specimens compared with aged specimens (*p* < 0.05). A similar trend was observed in the chlorhexidine group, in which non-aged specimens exhibited significantly greater bond strength than aged specimens under both etch-and-rinse (*p* < 0.05) and self-etch (*p* < 0.05) conditions. In the resin-extract group, non-aged specimens also demonstrated significantly greater bond strength than aged specimens under both etch-and-rinse (*p* < 0.05) and self-etch (*p* < 0.05) surface preparation methods.

Agents used as cavity disinfectants are expected to exhibit strong antibacterial activity while not negatively affecting the adhesion of restorative materials to dental substrates. Therefore, evaluating the effects of these agents on the dentin-adhesive interface is of great importance. Thermal cycling and aging tests, which examine the interaction between the adhesive system and the dentin substrate in order to simulate the dynamic conditions of the oral environment, are frequently used in laboratory studies [25]. The application time and duration of cavity disinfectants can play a decisive role in bond strength. In etch-and-rinse adhesive systems, it is recommended to apply disinfectants after etching due to the resistance of the dentin surface to the etching process; however, in self-etch systems, it is reported that applying disinfectants before etching is more appropriate [25]. The most commonly reported application time for chlorhexidine in the literature is 60 s. In addition, it was demonstrated that even short-term chlorhexidine applications slowed down the degradation occurring at the dentin-resin interface [26]. Accordingly, in the present study, an application time of 60 s was preferred, consistent with the literature, and the recommended application sequence specific to adhesive systems was followed.

Following the shear bond strength test, the fracture modes of the specimens were examined under a stereomicroscope at 40× magnification, and their distribution is presented in Table 6. Mixed-type fractures were the most prevalent across all groups. No statistically significant differences in fracture modes were observed among the cavity disinfectant groups for either aged or non-aged specimens under both self-etch and etch-and-rinse surface preparation methods (*p* > 0.05). The calculated values for all groups ranged from 0.784 to 0.982, which are above the commonly accepted significance level of 0.05 (*p* > 0.05). These results indicate that there were no statistically significant differences in the distribution of fracture modes among the cavity disinfectants (Control, Chlorhexidine, and Resin Extract), regardless of the aging process or the surface preparation method applied.

In this study, the initial bond strengths of the control, chlorhexidine, and *P. orientalis* groups were compared with the values obtained after a 6-month thermal cycle. A statistically significant decrease in bond strength was observed in all groups after the aging process (*p* < 0.05). However, no significant difference was found in bond strength between the *P. orientalis* and chlorhexidine groups, and it is thought that *P. orientalis* resin could be a potential alternative disinfectant in terms of maintaining adhesive interface stability.

One of the main causes of degradation occurring over time at the adhesive interface is the activation of endogenous matrix metalloproteinases (MMPs) found in the dentin collagen matrix. It has also been reported that MMP activity increases, especially after etching, and that this accelerates the hydrolytic degradation of the hybrid layer. Chlorhexidine, thanks to its MMP inhibitory effect, supports the long-term stability of the hybrid layer and is considered the “gold standard” cavity disinfectant in the literature [27,28,29]. Indeed, previous studies have shown that the increased MMP activity in dentin after etching can be effectively suppressed with inhibitory agents such as chlorhexidine [28]. In the present study, the fact that the *P. orientalis* group showed similar bond strength results with chlorhexidine suggests that this agent may be effective on adhesive interface degradation through similar biochemical mechanisms.

Although many studies have reported that chlorhexidine can better maintain post-aging bond strength thanks to its MMP inhibitory effect [28,29], some studies have also reported that this effect is limited or not statistically significant [30,31,32]. In the present study, a decrease in post-aging bond strength was observed in both etch-and-rinse and self-etch systems; however, the decrease in the chlorhexidine group was determined to be lower compared to the control group. In the evaluation of natural and resin-derived alternatives, MMP inhibition potential, similar to that of chlorhexidine, stands out as an important criterion. *P. orientalis* extracts may have the potential to offer similar MMP inhibitory activity due to their high caffeic acid content, as caffeic acid and its derivatives can block the MMP catalytic site [33,34,35]. Furthermore, ethanol solvent was known to have a suppressive effect on MMP-9 activity [36]. These factors are considered together; it is thought that *P. orientalis* resin can maintain adhesive interface stability at a level close to that of chlorhexidine.

These findings suggest that *P. orientalis* extract can maintain adhesive interface stability, highlighting its potential as a natural alternative to chlorhexidine. Similar to our study, a previous investigation using propolis demonstrated that it acts as an effective cavity disinfectant that does not compromise dentin bonding and exhibits strong activity against specific bacteria such as S. aureus, although its spectrum is generally narrower than that of ClO_2_. Pine resin, which contains a comparable phenolic composition to propolis, showed a similar effect [37]. Studies with propolis extracts have also demonstrated that these extracts, similar to chlorhexidine, exhibit antimicrobial efficacy against specific oral microorganisms [37,38,39].

The present study demonstrated that *P. orientalis* resin exhibits notable antibacterial activity. Although propolis and *Pistacia lentiscus* (mastic gum) were not included as comparative materials, both resins are well documented for their antibacterial, antioxidant, and anti-inflammatory properties, particularly in dental applications. Propolis has been extensively investigated for its use in oral health, while mastic gum has been reported to be effective against key oral pathogens such as Streptococcus mutans and Lactobacillus species, contributing to the prevention of dental caries [9,10].

Considering the antibacterial efficacy reported for these natural resins, the findings of the present study suggest that *P. orientalis* resin may possess comparable bioactive potential. In this context, the high phenolic content and pronounced antibacterial activity observed indicate that *P. orientalis* resin could be considered a promising natural candidate for use as a cavity disinfectant. Nevertheless, further studies are required to directly compare its efficacy with established natural resins, as well as to clarify its antibacterial spectrum, mechanisms of action, and long-term biocompatibility under clinically relevant conditions.

## 3. Materials and Methods

### 3.1. Samples and Extractions

**Figure 2:**
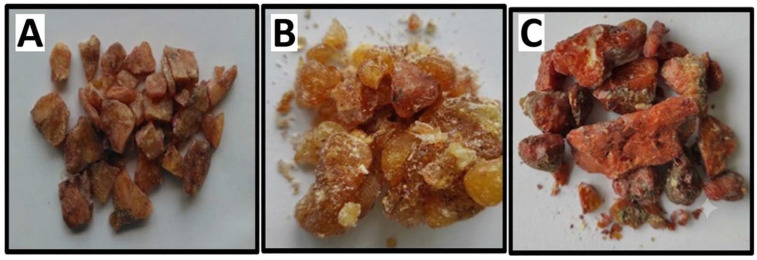
The resin of the *P. orientalis* samples (**A**) Artvin, (**B**) Borçka, (**C**) Çaykara.

In this study, three resin samples were collected during the autumn months (October–November) from the forested areas of the Artvin, Çaykara, and Borçka districts in the Black Sea region. The samples were obtained by scraping the trunks of *P. orientalis* trees and were subsequently stored at −20 °C to prevent degradation prior to analysis. The macroscopic appearance of the resin specimens is shown in Figure 2.

*P. orientalis* resin samples, stored at −20 °C, were ground into a fine powder using a porcelain mortar due to the limited quantity and adhesive nature of the resin samples.

A 3.5 g portion of the powdered resin was combined with 35 mL of 70% ethanol (Sigma-Aldrich, Steinheim, Germany) and shaken at 200 rpm at room temperature for 24 h. After extraction, the mixtures were filtered sequentially through standard filter paper followed by blue ribbon filter paper. A portion of the resulting extract was reserved for antioxidant assays and phenolic content analysis, while the remaining extract was stored at 4 °C for microbiological and shear bond strength testing [15].

### 3.2. Total Phenolic and Flavonoid Contents (TPC)

The total phenolic content was determined using the Folin–Ciocalteu assay [40], with gallic acid serving as the reference standard. To determine the total phenolic content, 40 µL of the sample extract was mixed with 0.40 mL of 0.2 N Folin–Ciocalteu reagent (Sigma-Aldrich, Steinheim, Germany) and 0.04 mL of a 10% Na_2_CO_3_ solution. The mixture was allowed to stand at room temperature for 120 min, after which the absorbance was measured at 760 nm. A calibration curve was generated using gallic acid concentrations ranging from 0.015 to 1.0 mg/mL (y=1.1510x +0.030, R2=0.999). TPC results were reported as mg GAE/g.

### 3.3. Total Flavonoid Contents (TPC)

The total flavonoid content (TFC) was determined according to method [41]. Using a quercetin standard calibration curve, the TFC was calculated and expressed as milligrams of quercetin (Sigma-Aldrich, Steinheim, Germany) equivalents per gram of sample (mg QE/g). To conduct this determination, 50 µL of the extract, 50 µL of 10% Al(NO_3_)_3 _ (Merck, Darmstadt, Germany) and 50 µL of 1.0 M ammonium acetate (NH_4_CH_3_COO) (Sigma-Aldrich, Steinheim, Germany) were sequentially mixed. The total volume was adjusted to 3.0 mL with 99% ethanol. The mixture was allowed to react for 45 min at 25 °C prior to measuring the absorbance at 415 nm. The results were calculated and derived from a quercetin standard curve and expressed as mg QE/g (y=0.6596x+0.001, R2=0.9983). All measurements were performed in triplicate, and the results were expressed as the mean values of three independent determinations.

### 3.4. Determination of FRAP Assay

The total antioxidant capacity was evaluated using the FRAP assay, in which antioxidants present in the sample reduce the Ferric–TPTZ complex (Fe^3+^–TPTZ) (Sigma-Aldrich, Steinheim, Germany) to its ferrous form (Fe^2+^–TPTZ) [42]. The working FRAP solution was freshly prepared by mixing 300 mM acetate buffer (pH 3.6), 10 mM TPTZ, and 20 mM FeCl_3_ in a 10:1:1 (*v*/*v*/*v*) ratio. For the assay, 50 µL of the tea infusion was combined with 1.50 mL of this FRAP reagent. The extent of this reduction was quantified using a calibration curve prepared with FeSO_4_·7H_2_O standards over a concentration range of 31.25–1000 µM (y = 0.0006x + 0.0127, R2 = 0.9995).

### 3.5. Determination of DPPH Radical-Scavenging Activity

The DPPH radical-scavenging activity of the extract was evaluated spectrophotometrically [43]. Briefly, 0.75 mL of 100 µM DPPH (Sigma-Aldrich, Steinheim, Germany) solution was mixed with 0.75 mL of the extract and incubated in the dark at 25 °C for 50 min. Absorbance was measured at 517 nm. To determine the SC_50_ value, six concentrations of the ethanolic extract were tested under identical conditions, and a dose–response curve was generated from the absorbance data. SC_50_ was expressed in mg/mL.

### 3.6. Determination of Phenolic Compositions

Phenolic compounds were quantified using an HPLC–PDA system (Shimadzu LC-20AT, Nakagyo-ku, Japan) equipped with a C18 column (250 × 4.6 mm, 5 µm). A calibration curve was generated from 26 phenolic standards. The mobile phase consisted of acetonitrile–water (70:30, *v*/*v*) as solvent A and 2% acetic acid as solvent B. Both standards and samples were injected at 20 µL, with a column temperature of 30 °C and a flow rate of 1.0 mL/min [44].

In this study, liquid–liquid extraction was applied to improve the identification of phenolic compounds in *P. orientalis* resin extract. Briefly, 10 mL of the methanolic extract was concentrated at 40 °C using a rotary evaporator (IKA®-Werke, RV 05 Basic, Staufen, Germany), and the residue was re-dissolved in 10 mL of water, with the pH adjusted to 2. The sample was then subjected to three successive extractions with diethyl ether and ethyl acetate. After the organic phases were evaporated, the remaining fraction was dissolved in 2 mL of methanol, filtered (0.45 µm RC), and analyzed by HPLC [16]. The results presented in Table 2 were calculated and expressed as µg/g.

Identification of the phenolic compounds was carried out by comparing the retention times and UV–Vis spectral characteristics of the detected peaks with those of corresponding reference standards. In addition, peak assignments were further confirmed by spiking experiments (standard addition), where co-injection of selected standards resulted in a proportional increase in peak area without changes in retention time, supporting accurate compound identification.

### 3.7. Antibacterial Activities

The antibacterial activity of the resin extracts was evaluated using the agar well diffusion method. Standard bacterial strains of *Staphylococcus aureus* ATCC 25923, *Enterococcus faecalis* ATCC 29212, *Pseudomonas aeruginosa* ATCC 10145, and *Streptococcus mutans* RSKK 07038 were revived from −80 °C stocks on Mueller–Hinton agar (MHA) and incubated at 37 °C for 18–24 h. Fungal strains (*Candida albicans* ATCC 10231 and *Candida parapsilosis* ATCC 22019) were cultured on potato dextrose agar (PDA) [17].

Bacterial suspensions were adjusted to 0.5 McFarland in phosphate-buffered saline, while *Candida* spp. were standardized to 1 McFarland and inoculated onto MHA supplemented with 2% glucose and methylene blue (Alfa Aeser, Ward Hill, MA, USA). After lawn inoculation using sterile swabs, 6 mm wells were punched into the agar. Test samples (50 µL) were pipetted into each well. Ampicillin and gentamicin served as positive controls for Gram-positive and Gram-negative bacteria, respectively, while amphotericin B was used for yeasts.

Antibacterial activity was determined by measuring inhibition zone diameters around the wells. All assays were performed in triplicate. Activity levels were classified as follows: <6 mm (inactive), 7–9 mm (very low), 9–11 mm (low), 12–14 mm (moderate), and >15 mm (high). 

#### Determination of MICs and MBCs

The minimum inhibitory concentrations (MICs) and minimum bactericidal concentrations (MBCs) were determined for compounds that exhibited antibacterial activity in the agar well diffusion assay. For MIC testing, 96-well U-bottom microplates were prepared by adding 100 µL of MHB-II medium to each well, followed by 100 µL of the test compound to the first well of each row. Serial two-fold dilutions were then performed. Positive control wells received 100 µL of antibiotics at an initial concentration of 200 µg/mL, followed by the same dilution procedure. Subsequently, 5 × 10^5^ CFU/mL of the test microorganisms were inoculated into each well. The lowest concentration showing no visible growth was recorded as the MIC.

To determine the MBC, 50 µL from wells at the MIC and the preceding higher concentration were spread onto agar plates. After incubation, the lowest concentration yielding no colony growth on agar was designated as the MBC [21].

### 3.8. Shear Bond Strength Test

#### 3.8.1. Sample Preparation

This study was approved by the Ethical Research Committee of the Karadeniz Technical University (2020/339). A total of 180 caries-free, fully developed, unerupted human third molars extracted within the last six months for orthodontic, surgical, or other oral reasons were selected. Teeth with cracks or extraction trauma were excluded. Soft tissue and bone residues were removed with a periodontal curette, and teeth were stored at +4 °C in 0.1% thymol solution for one week, then kept in distilled water with weekly renewal [13].

All specimens were fixed with auto-polymerizing acrylic (Imicryl, SC, Konya, Turkey), leaving one-third of the root exposed. The occlusal enamel was removed to expose mid-coronal dentin. Parallel dentin sections were obtained under water-cooling with a low-speed precision cutter (Micracut 125, Low Speed Precision Cutter, Metkon, Bursa, Türkiye) with a 0.5 mm diamond blade (DIMOS Ø150 mm, Metkon, Türkiye).

Dentin samples were embedded in 15 mm diameter × 8 mm height plastic molds compatible with the testing device. Exposed dentin surfaces were polished under running water with 600-grit sandpaper (Rhynowet Red Line, Aveiro, Portugal) for 60 s to create a standardized smear layer and flat dentin surface. Sandpaper was replaced after every 10 teeth to maintain consistency.

#### 3.8.2. Grouping and Restorative Procedures

The 180 dentin specimens were randomly divided into three groups based on the cavity disinfectant: control, 2% chlorhexidine gluconate (Ceraxidin-C, Imicryl), and Artvin *P. orientalis* resin extract (selected for highest antibacterial activity). Each group was further subdivided into two subgroups according to the adhesive strategy: self-etch or etch-and-rinse. For the etch-and-rinse subgroups, 37% phosphoric acid (Condac 37, FGM, Joinville, Brazil)) was applied for 15 s prior to adhesive placement. All specimens were restored with a universal adhesive (Single Bond Universal, 3M ESPE, Seefeld, Germany) and composite resin (Estelite Sigma Quick, Tokuyama Dental, Tokyo, Japan) using a transparent mold (3 mm diameter × 4 mm height). Polymerization was performed with an LED light source (Elipar Freelight, 3M ESPE). Specimens in each subgroup were further divided into two aging conditions to evaluate short- and long-term bond strength, with 15 dentin samples per subgroup (n = 15). Study groups are summarized in Table 4.

#### 3.8.3. Thermal Cycling Aging

Specimens assigned for aging were subjected to 5000 thermal cycles between 5 °C and 55 °C (±2 °C) to simulate oral temperature fluctuations. Each cycle included a 30 s dwell time in the baths and a 2 s transfer time between them, using a thermal cycling device (Nova, Konya, Türkiye). This procedure approximates six months of clinical service [22].

#### 3.8.4. Shear Bond Strength Testing and Failure Mode Analysis

Dentin specimens were either stored at 37 °C and 100% relative humidity for 24 h (non-aged) or subjected to thermal cycling (5–55 °C, 5000 cycles) prior to testing. Shear bond strength (SBS) was measured using a Universal Testing Machine (MTS Criterion, Minneapolis, MN, USA). Specimens were secured in a custom metal jig, and a knife-edge blade applied force at a crosshead speed of 0.5 mm/min at a 90° angle to the adhesive interface. The peak load at failure was recorded in Newtons (N) and converted to megapascals (MPa) by dividing by the bonded area (MPa = N/mm^2^). Failure modes were evaluated under a stereo microscope (Leica, Wetzlar, Germany) at ×40 magnification and classified as adhesive, cohesive, or mixed, as summarized in Table 5. Adhesive failure involved complete separation at the composite–dentin interface; cohesive failure occurred within either the dentin or composite; mixed failure included combinations of adhesive and cohesive failures within dentin or composite, or partial adhesive failure with cohesive involvement.

### 3.9. Statistical Analysis

Shear bond strength data were analyzed using IBM SPSS Statistics 22. Normality was confirmed with the Kolmogorov–Smirnov and Shapiro–Wilk tests. The effects of cavity disinfectants, adhesive strategy, and aging on bond strength were evaluated by three-way ANOVA and post hoc Tukey’s HSD post hoc test. Failure mode distributions were analyzed using the Chi-square test. Significance was set at *p* < 0.05.

## 4. Conclusions

The present study demonstrates that *P. orientalis* resin extracts exhibit potent antioxidant capacity and significant targeted antibacterial efficacy against *S. aureus*. Crucially for restorative dentistry, cavity pre-treatment with these natural extracts preserved dentin adhesive bond strength following aging protocols, performing comparably to the clinical gold standard, chlorhexidine. The synergistic effect of its primary phenolic constituents (caffeic, ferulic, and trans-cinnamic acids), combined with the matrix metalloproteinase (MMP) inhibitory potential of the ethanolic solvent, underscores the substantial potential of *P. orientalis* resin as a highly effective, biocompatible natural cavity disinfectant. While these findings strongly position this pine resin as a promising botanical antibacterial agent for dental applications, future longitudinal in vivo studies are required to comprehensively validate its long-term clinical safety and therapeutic performance.

## Figures and Tables

**Table 1:** Total phenolic content and antioxidant properties of the *P. orientalis* extracts.

	Artvin (n:3)	Borçka (n:3)	Çaykara (n:3)
Total phenolic content (mg GAE/g)	389.47 ± 5.64 ^a^	387.66 ± 5.17 ^a^	378.29 ± 5.87 ^a^
Total flavonoid content (mg QE/g)	2.97 ± 0.07 ^a^	0.09 ± 0.01 ^b^	0.11 ± 0.01 ^b^
FRAP (μmoL FeSO4.7H2O/g)	3312.69 ± 32.32 ^a^	3646.69 ± 16.03 ^a^	2955.52 ± 12.01 ^b^
DPPH SC_50_ (µg/mL)	40.00 ± 0.50 ^a^	20.02 ± 0.10 ^a^	30.00 ± 2.05 ^a^

The values in the table are given as average values. Letters in each cell indicate differences at *p* < 0.05 (one-way ANOVA test) level.

**Table 2:** Phenolic composition of *Picea orientalis* extracts.

[µg/g]	Artvin (n:3)	Borçka (n:3)	Çaykara (n:3)
*p*-OH Benzoic acid	18.06 ± 1.02	>LOD	>LOD
Caffeic acid	1664.26 ± 12.56 ^a^	1255.84 ± 125.98 ^b^	1354.07 ± 35.90 ^c^
Ferulic acid	347.96 ± 32.22 ^a^	300.30 ± 32.21 ^a^	360.65 ± 9.45 ^a^
*t*-Cinnamic acid	5948.39 ± 182.02 ^a^	7451.05 ± 567.60 ^b^	5753.25 ± 462.09 ^a^
Epicatechin	118.69 ± 4.33	>LOD	>LOD
Hesperetin	874.26 ± 65.70	-	>LOD

Different lowercase letters within columns indicate statistically significant differences among cavity disinfectants and surface preparation methods, respectively (*p* < 0.05).

**Table 3:** Antibacterial activity of samples and some phenolic standards using Minimum Inhibitory Concentration (MIC) and Minimum Bactericidal Concentration (MBC).

	*S. aureus* (+)			*E. faecalis* (+)			*P. aeruginosa* (−)		
	Zone	MIC	MBK	Zone	MIC	MBC	Zone	MIC	MBC
**Artvin**	18.60 ± 0.57	125	250	-	-	-	-	-	-
**Çaykara**	16.30 ± 0.00	250	500	-	-	-	-	-	-
**Borçka**	18.66 ± 0.57	250	500	-	-	-	-	-	-
**Caffeic acid**	22.00 ± 0.00	62.5	125	12.00 ± 0.00	500	1000	14.00 ± 0.00	500	1000
**Ferulic acid**	-	-	-	-	-	-	-	-	-
**t-cinnamic acid**	-	-	-	-	-	-	-	-	-
**Chlorhexidine**	26.00 ± 1.00	15.6	31.25	23.6 ± 2.08	62.5	125	22.00 ± 0.00	62.5	125
**Ampicillin**	33.00 ± 0.00	7.81	15.6	27.33 ± 0.57	31.25	62.5	-	-	-
**Gentamicin**	-	-	-	-	-	-	29.33 ± 0.57	15.6	31.25
**Amphotericin B**	-	-	-	-	-	-	-	-	-

Zone (mm), MIC (µg/mL), ≤14; Low level of activity, ≥15 and ≤24; Moderate level of activity, ≥25; High activity.

**Table 4:** Antibacterial activity of samples and some phenolic standards using Minimum Inhibitory Concentration (MIC) and Minimum Bactericidal Concentration (MBC).

	*S. mutans* (−)				*C. albicans*			*C. parapsilosis*		
	Zone	MIC	MBK	Zone		MIC	MBK	MBC	Zone	MIC
**Artvin**	8.00 ± 0.00	500	1000	-		-	-	-	-	-
**Çaykara**	10.00 ± 0.00	500	1000	-		-	-	-	-	-
**Borçka**	8.00 ± 0.50	500	1000	-		-	-	-	-	-
**Caffeic acid**	11.50 ± 0.50	500	1000	-		-	-	-	-	-
**Ferulic acid**	-	-	-	-		-	-	-	-	-
**t-cinnamic acid**	-	-	-	-		-	-	-	-	-
**Chlorhexidine**	35.00 ± 0.00	7.81	15.6	25.00 ± 0.00		15.6	31.25	22.33 ± 0.57	15.6	31.35
**Ampicillin**	30.00 ± 0.00	15.6	31.25	-		-	-	-	-	-
**Gentamicin**	-	-	-	-		-	-	-	-	-
**Amphotericin B**	-	-	-	29.00 ± 0.00		7.81	15.6	27.33 ± 0.57	15.6	31.25

Zone (mm), MIC (µg/mL), ≤14; Low level of activity, ≥15 and ≤24; Moderate level of activity, ≥25; High activity.

**Table 5:** Evaluation of the effects of aging and different surface preparation methods on the bond strength of cavity disinfectants.

Aging Status	Surface Preparation Method	Control (Mean± SD)	Chlorhexidine (Mean ± SD)	Resin Extract (Mean ± SD)
Initial	Self-etch	21.25 ± 2.23 ^Aa^	20.59 ± 2.03 ^Aa^	20.19 ± 2.34 ^Aa^
	Etch & Rinse	25.67 ± 1.18 ^Ab^	23.82 ± 1.65 ^Ba^	22.6 ± 2.50 ^Ba^
Aged	Self-etch	17.23 ± 2.06 ^Aa^	19.91 ± 1.92 ^Ba^	19.41 ± 2.61 ^Ba^
	Etch & Rinse	19.69 ± 1.57 ^Ab^	20.04 ± 3.10 ^Aa^	20.53 ± 2.51 ^Aa^

Different uppercase letters in the rows indicate significant differences among the cavity disinfectants. Different lowercase letters in the columns indicate significant differences among the surface preparation methods.

**Table 6:** Effect of Cavity disinfectants on fracture mode distribution in aged and non-aged dentin bonded with self-etch and etch-and-rinse systems.

Aging	Surface Preparation Method	Fracture Type	Control	Chlorhexidine	Resin Extract	*p*-Value (Chi-Square Test)
Initial	Self-etch	Adhesive	6 (40.0%)	7 (46.7%)	6 (40.0%)	0.893
		Cohesive Composite	1 (6.7%)	0 (0.0%)	1 (6.7%)	
		Mixed	8 (53.3%)	8 (53.3%)	8 (53.3%)	
	Etch & Rinse	Adhesive	6 (40.0%)	6 (40.0%)	6 (40.0%)	0.982
		Cohesive Dentin	1 (6.7%)	1 (6.7%)	1 (6.7%)	
		Cohesive Composite	1 (6.7%)	1 (6.7%)	0 (0.0%)	
		Mixed	7 (46.7%)	7 (46.7%)	8 (53.3%)	
Aged	Self-etch	Adhesive	7 (46.7%)	7 (46.7%)	6 (40.0%)	0.914
		Mixed	8 (53.3%)	8 (53.3%)	9 (60.0%)	
	Etch & Rinse	Adhesive	6 (40.0%)	6 (40.0%)	7 (46.7%)	0.784
		Cohesive Dentin	0 (0.0%)	1 (6.7%)	0 (0.0%)	
		Cohesive Composite	1 (6.7%)	0 (0.0%)	1 (6.7%)	
		Mixed	8 (53.3%)	8 (53.3%)	7 (46.7%)	

## Data Availability

The datasets were generated or analyzed during the current study. The raw data supporting the conclusions of this article will be made available by the authors on request.

## Supplementary Materials

The following supporting information can be downloaded at: https://www.mdpi.com/article/10.3390/molecules31142529/s1, S1. HPLC chromatogram of the resin extract from Artvin region, (4) p-OH benzoic acid, (5) Epicatechin, (6) Caffeic Acid, (12) Ferulic Acid, (18) t-Cinnamic acid, (20) Hesperetin. S2. HPLC chromatogram of the resin extract from Borçka region, (6) Caffeic Acid, (12) Ferulic Acid, (18) t-Cinnamic acid. S3. HPLC chromatogram of the resin extract from Çaykara region, (6) Caffeic Acid, (12) Ferulic Acid, (18) t-Cinnamic acid.

## Abbreviations

The following abbreviations are used in this manuscript:

ANOVA	One-way analysis of variance
DPPH•	2,2-diphenyl-1-picrylhydrazyl
CAPE	Caffeic Acid Phenethyl Ester
GAE	Gallic acid equivalents
FRAP	Ferric reducing antioxidant power
HPLC	High-pressure liquid chromatography
PDA	Photodiode array
QE	Quercetin equivalent
MMP	Matrix metalloproteinases
TPC	Total phenolic content
TFC	Total flavonoid content
TPTZ	2,4,6-tripyridyl-s-triazine
LOD	Limit of detection
MIC	Minimum inhibitory concentrations
MBC	Minimum bactericidal concentrations
