# Supplementary material for: Antioxidant, Antibacterial, and Dental Bond Strength Performance of Picea orientalis Resin
Source: Molecules. 2026 Jul 21;31(14):2529. doi: 10.3390/molecules31142529 (PMC13414595; doi:10.3390/molecules31142529)
Supplement: Supplementary file 1 [file molecules-31-02529-s001.zip › molecules-4349610-supplementary.pdf]

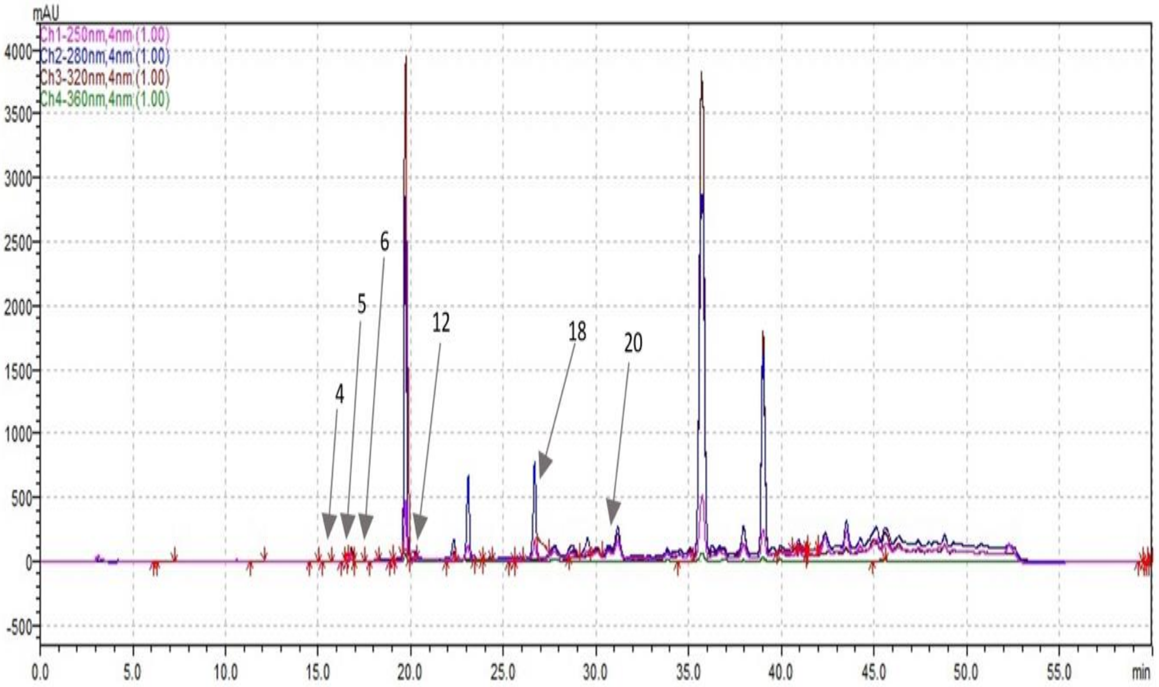

Supplementary Materials S1. HPLC chromatogram of the resin extract from Artvin region, (4) p-OH benzoic acid, (5) Epicatechin, (6) Caffeic Acid, (12) Ferulic Acid, (18) t-Cinnamic acid, (20) Hesperetin

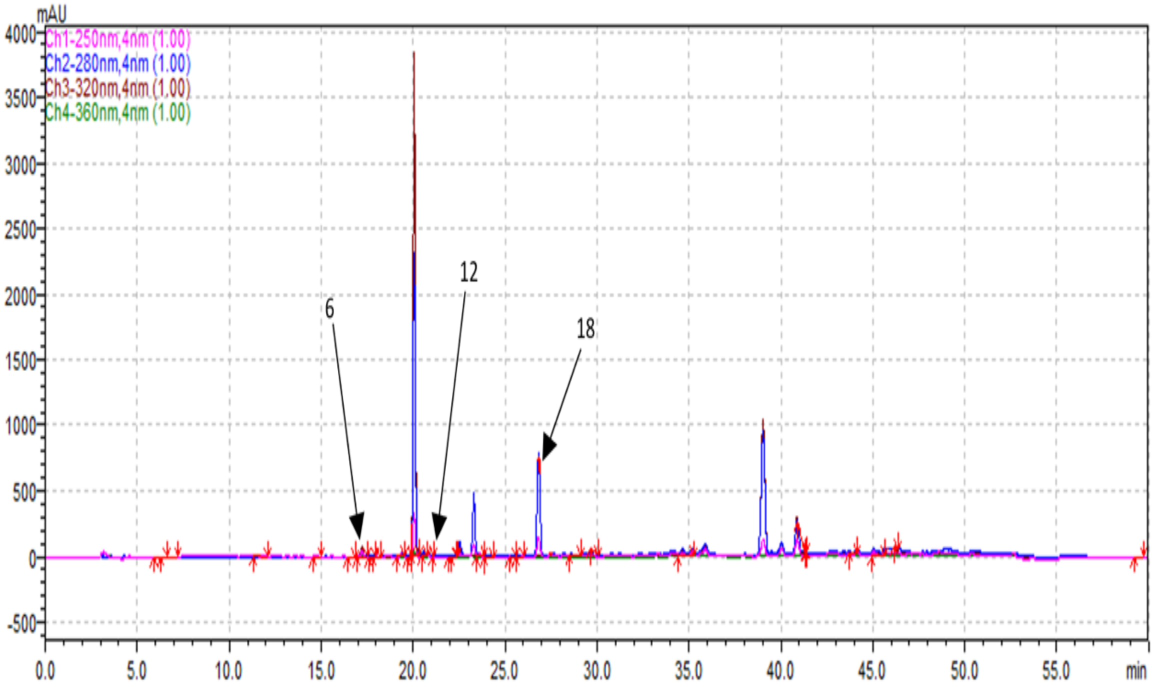

Supplementary Materials S2. HPLC chromatogram of the resin extract from Borçka region, (6) Caffeic Acid, (12) Ferulic Acid, (18) t-Cinnamic acid

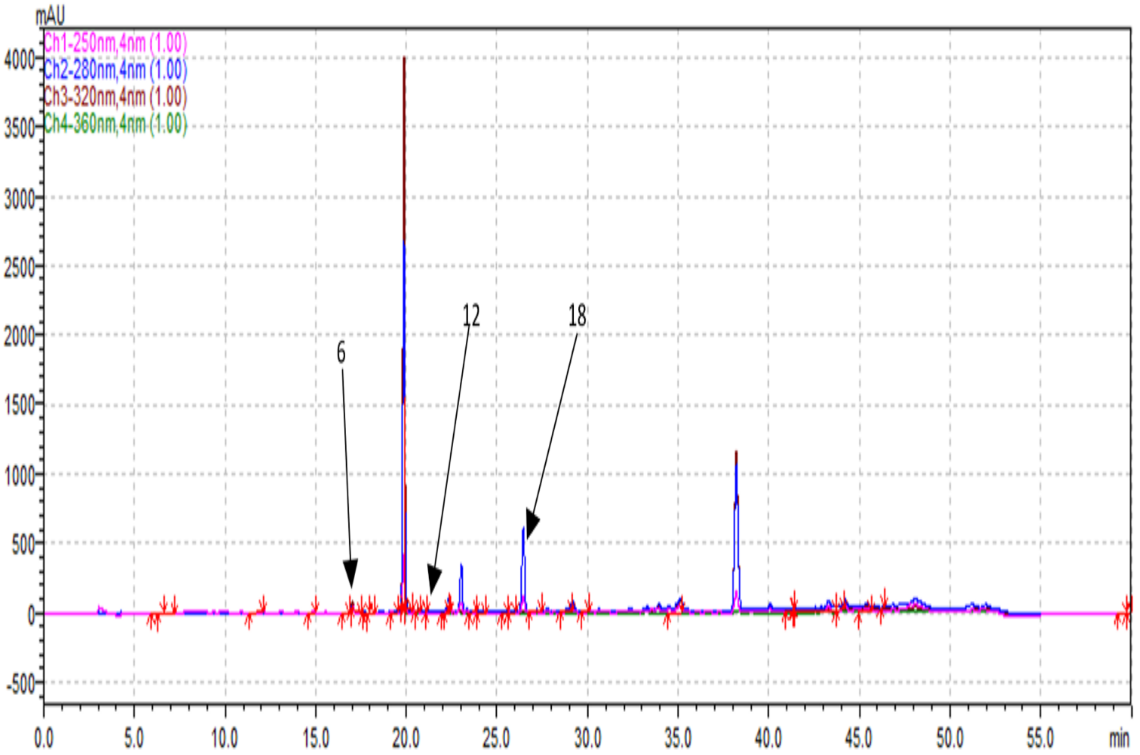

Supplementary Materials S3. HPLC chromatogram of the resin extract from Çaykara region, (6) Caffeic Acid, (12) Ferulic Acid, (18) t-Cinnamic acid
